# Toward point-of-care management of chronic respiratory conditions: Electrochemical sensing of nitrite content in exhaled breath condensate using reduced graphene oxide

**DOI:** 10.1038/micronano.2017.22

**Published:** 2017-05-22

**Authors:** Azam Gholizadeh, Damien Voiry, Clifford Weisel, Andrew Gow, Robert Laumbach, Howard Kipen, Manish Chhowalla, Mehdi Javanmard

**Affiliations:** 1 Department of Electrical and Computer Engineering, Rutgers University, Piscataway, NJ 08854, USA; 2 Department of Material Science and Engineering, Rutgers University, Piscataway, NJ 08854, USA; 3 Environmental Occupational Health Sciences Institute, Rutgers University, Piscataway, NJ 08854, USA; 4 School of Pharmacy, Rutgers University, Piscataway, NJ 08854, USA

**Keywords:** exhaled breath condensate, nitrite, electrochemistry, square wave voltammetry, thin-layer-reduced graphene oxide

## Abstract

We present a portable non-invasive approach for measuring indicators of inflammation and oxidative stress in the respiratory tract by quantifying a biomarker in exhaled breath condensate (EBC). We discuss the fabrication and characterization of a miniaturized electrochemical sensor for detecting nitrite content in EBC using reduced graphene oxide. The nitrite content in EBC has been demonstrated to be a promising biomarker of inflammation in the respiratory tract, particularly in asthma. We utilized the unique properties of reduced graphene oxide (rGO); specifically, the material is resilient to corrosion while exhibiting rapid electron transfer with electrolytes, thus allowing for highly sensitive electrochemical detection with minimal fouling. Our rGO sensor was housed in an electrochemical cell fabricated from polydimethyl siloxane (PDMS), which was necessary to analyze small EBC sample volumes. The sensor is capable of detecting nitrite at a low over-potential of 0.7 V with respect to an Ag/AgCl reference electrode. We characterized the performance of the sensors using standard nitrite/buffer solutions, nitrite spiked into EBC, and clinical EBC samples. The sensor demonstrated a sensitivity of 0.21 μA μM^−1^ cm^−2^ in the range of 20–100 μM and of 0.1 μA μM^−1^ cm^−2^ in the range of 100–1000 μM nitrite concentration and exhibited a low detection limit of 830 nM in the EBC matrix. To benchmark our platform, we tested our sensors using seven pre-characterized clinical EBC samples with concentrations ranging between 0.14 and 6.5 μM. This enzyme-free and label-free method of detecting biomarkers in EBC can pave the way for the development of portable breath analyzers for diagnosing and managing changes in respiratory inflammation and disease.

## Introduction

Biomarkers have enormous potential utility in assessing chronic inflammation, especially in asthma, which affects ~300 million people worldwide. Asthma, which is characterized by variable airway inflammation and air flow obstruction, is an increasingly important global health problem. In the United States alone, ~17.7 million adults and 6.3 million children were diagnosed with asthma in 2014 (Ref. 1). Furthermore, the cost of asthma care in the United States was estimated to be $56 billion in 2007. The currently available non-invasive methods for diagnosing and monitoring asthma, i.e., spirometry and the measurement of exhaled nitric oxide, are limited by low sensitivity and the need for expensive and bulky equipment. Moreover, existing tests have a limited ability to characterize the nature and extent of underlying airway inflammation, which is widely variable between individuals^2^. Measurement of biomarkers in exhaled breath condensate (EBC) can contribute to the molecular phenotyping of asthma, thus enabling targeted treatment and more effective disease management. Given the large and growing burden of asthma, there is an urgent need for improved, minimally invasive methods for the molecular diagnosis and monitoring of asthma.

The use of biomarkers in EBC may help to overcome the difficulties associated with obtaining airway tissue and bronchoalveolar lavage samples that have significantly hampered the study of naturally occurring exacerbations of asthma. EBC contains droplets of airway lining fluid (ALF) that are exhaled during normal tidal breathing. In addition to condensed gas-phase compounds, EBC contains non-volatile compounds that originate from ALF, including hydrogen peroxide, nitrite and nitrate, as well as larger molecules such as eicosanoids, proteins, and even nucleic acids^3–5^. The ability to non-invasively characterize airway tissue by repeated measurements of biomarkers in EBC would be invaluable for studying the time-course of dynamic inflammatory pathways that are involved in asthma exacerbation. Ultimately, EBC biomarkers may contribute to the assessment of different asthma phenotypes and the development of individualized rational approaches to asthma management at the point of care^6–9^.

**Figure 1:**
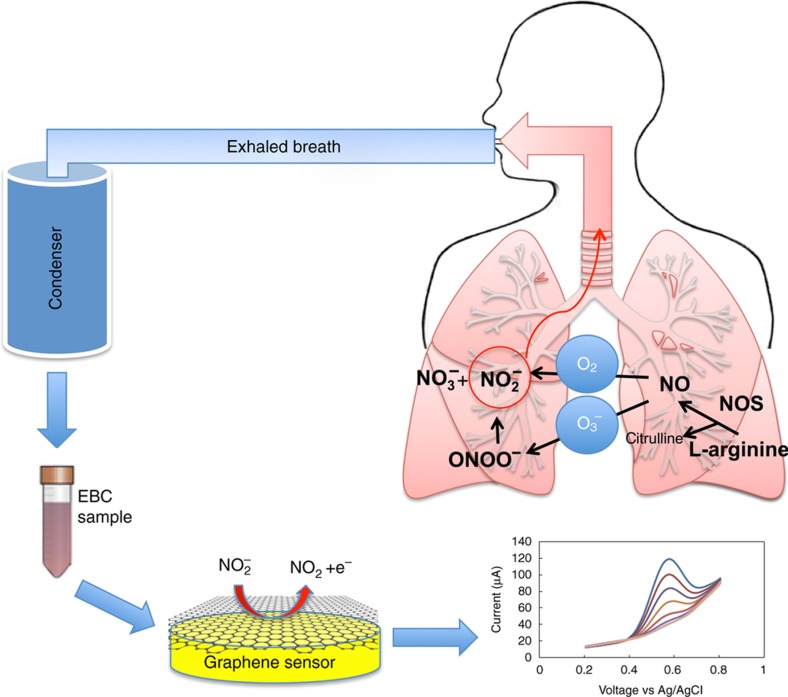
An exhaled breath condensate (EBC) sample is collected, and nitrite content is measured electrochemically.

Recent studies have shown the promise of EBC nitrite for use as a biomarker of both oxidative stress and inflammation in asthma (Figure 1). The primary source of nitrite in the respiratory tract is nitric oxide (NO), which is produced from L-arginine by nitric oxide synthase. In aqueous solution, NO reacts rapidly with reactive oxygen species (ROS) to form more stable nitrogen oxides, such as nitrite (NO^−^_2_) and nitrate (NO^−^_3_)^10^. Increased levels of NO are associated with inflammatory disease states such as asthma, COPD^11^, and cystic fibrosis^12,13^. The increased level of exhaled NO in asthma has been suggested to be due to an increased expression of inducible NO synthase (iNOS) in bronchial epithelium^14^. Given the relative stability of nitrite in EBC and its promise as a biomarker of chronic respiratory inflammation, we developed a miniaturized probe-free/label-free sensor for the detection of nitrite in EBC.

Nitrite is typically detected through one of several spectrophotometric methods (Griess reaction) involving fluorimetry, chemiluminescence, or ion chromatography^15–28^. The detection limit of fluorimetric methods is 0.1 μM. Chemiluminescence has a lower detection limit (in the nM range). Nitrite concentrations in EBC are in the μM range and are compatible with these detection limits. However, despite the low detection limit provided by these methods, EBC samples are usually pretreated to induce the appropriate reaction and/or to eliminate interfering compounds, such as chlorine^29^. The benefit of using electrochemical methods is that pretreatment steps are not required; more importantly, both the sensor and the instrumentation readout can be readily miniaturized, thus enabling the development of point-of-care diagnostics and even real-time wearable health-monitoring devices. The other strong benefit of using electrochemical methods is that they can be used to detect nitrite content at a specific potential of 0.7 V in real time without interference from other compounds in the EBC matrix. The drawbacks, however, are that these methods often do not offer the same detection limit as those provided by optical methods, and standard electrochemical cells require sample volumes of several milliliters.

The electrochemical detection of nitrite is based on either the oxidation or reduction of nitrite^30–32^. Nitrite oxidation-based methods with a final product of NO_3_ are usually preferred because the presence of interfering molecules (such as oxygen) during reduction can be avoided^33^. However, an oxidation reaction as the basis for detecting nitrite requires a high over-potential^34^; thus, in recent years, many attempts have been made to develop novel electrode materials^35–48^.

Among possible novel materials, graphene-based electrodes have been widely used due to their low over-potentials, resilience against corrosion, low electron transfer resistance, small residue currents, wide potential window, excellent chemical stability and potential for chemical functionalization. Graphene has previously been explored to detect a wide range of chemical and biological species^49–52^. Here, we used graphene-based electrodes to detect nitrite in EBC. A nitrite detector was fabricated using screen-printed electrodes that were modified with electrochemically reduced graphene oxide. Another limiting factor for graphene-based electrochemical sensors is that simple drop cast methods are used to deposit graphene oxide on a metal electrode film; this method can result in device-to-device variation due to the agglomeration of graphene oxide flakes. To resolve this issue, we used a modified drop cast method that results in thinner and precisely patterned rGO electrodes. We also fabricated a micro-electrochemical cell using a polydimethyl siloxane (PDMS) membrane to enable voltammetric measurements of very small sample volumes, which is necessary for analyzing EBC samples, where limited sample volumes are available. To the best of our knowledge, this represents the first electrochemical sensor that can directly measure nitrite content in clinical EBC samples with submicromolar detection limits.

## Materials and methods

### Sample preparation

EBC samples collected for a previous study^53^ were utilized. The details of the sample collection procedure have been previously described, but briefly, 1–2 mL of EBC was collected during 20 min of tidal breathing from each of seven adult subjects using an EcoScreen device (Jaeger, Wurzburg, Germany), which condensed the exhaled breath at −20 °C. All surfaces were triple-rinsed with nitrite-free water prior to contacting the EBC, and the samples were frozen at −80 °C for later analysis. In the previous study, nitrite concentrations were measured using selective catalytic reduction and chemiluminescence detection (NOA 280i, GE Analytics, Boulder, CO, USA)^53^. All solutions were prepared with distilled water. For testing and calibration of the sensors, we experimented with various buffers, including sodium nitrite, acetate, and phosphate buffers (Sigma-Aldrich, St Louis, MO, USA).

### Sensor fabrication and characterization

Graphene oxide was prepared using the Hummers method^54^. Screen-printed three-electrode micro-chips consisting of Ag/AgCl reference electrodes, platinum counter electrodes, and 5-mm gold working electrodes were commercially obtained (Metrohm, Herisau, Switzerland)^54^. The morphology of the graphene oxide was characterized using field-emission scanning electron microscopy (SEM) (Zeiss leo Field emission SEM, Carl Zeiss, Inc., One Zeiss Drive, NY, USA) and atomic force microscopy (AFM) (Digital Instruments Nanoscope IV, Digital Instruments, NY, USA). The atomic force microscope was operated in tapping mode using standard cantilevers with a spring constant of 40 N m^−1^ and a tip curvature of <10 nm. FT-Raman spectra (Horiba Johin-Yvon Micro Raman Spectrometer, 532 nm excitation laser, HORIBA, NY, USA) were recorded to characterize the reduction of the graphene oxide substrates. Electrochemical measurements (PSTAT Princeton Instruments, Trenton, NJ, USA) were performed under ambient conditions. All potentials were applied with respect to the Ag/AgCl reference electrode.

**Figure 2:**
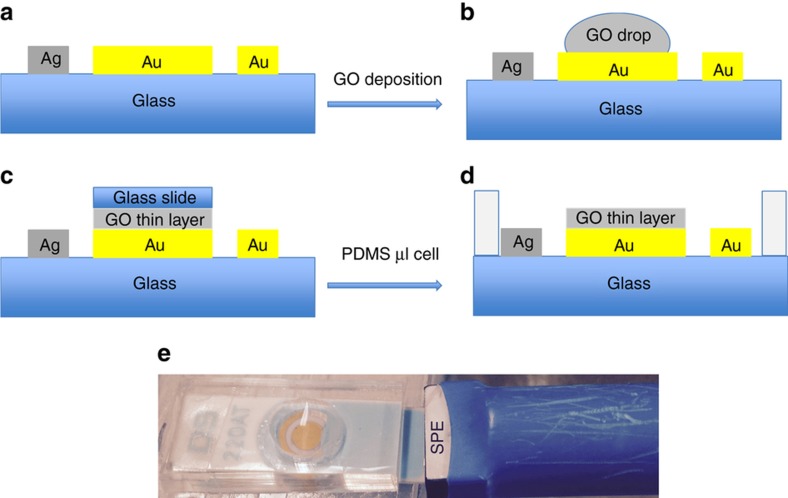
Fabrication process for the reduced graphene oxide biosensor formed in a micro-electrochemical fluidic cell. (**a**) shows electrodes of screen printed electrodes and (**b**) shows dropping 2 micro-litter graphene oxide solution on top of gold working electrode, creating thin layer graphene oxide layer indicates in part (**c**), part (**d**) shows how we can have small volume area with PDMS membrane and part (**e**) shows image of sensor we used for detection nitrite.

The steps used to fabricate the integrated reduced graphene oxide electrode/micro-electrochemical cell are shown in Figure 2. First, a 3-μL aliquot of graphene oxide suspension, which was synthesized from graphite powders using the Hummers method, was placed on the gold electrode surface. Then, a thin glass slide was placed on top of the droplet to cast the GO onto the gold electrode. Superfluous solution was aspirated, and the surface was dried at room temperature. The GO layer was then reduced electrochemically in acetate buffer (pH 5.5) using cyclic voltammetry between −1.6 and 0 V at a scan rate of 25 mV for 30 cycles under continuous N_2_ purging. The micro-electrochemical cell was fabricated by forming a thin layer of PDMS on top of the insulated layers of the SPE micro-chip. Then, a thicker PDMS layer containing an 8-mm hole was covalently bonded to the thin PDMS layer using O_2_ plasma treatment. During O_2_ plasma treatment, the rGO layer and the wire-bonding pads of the SPE micro-chip were protected with a glass slide.

## Results and discussion

### Characterization of graphene oxide sensors

**Figure 3:**
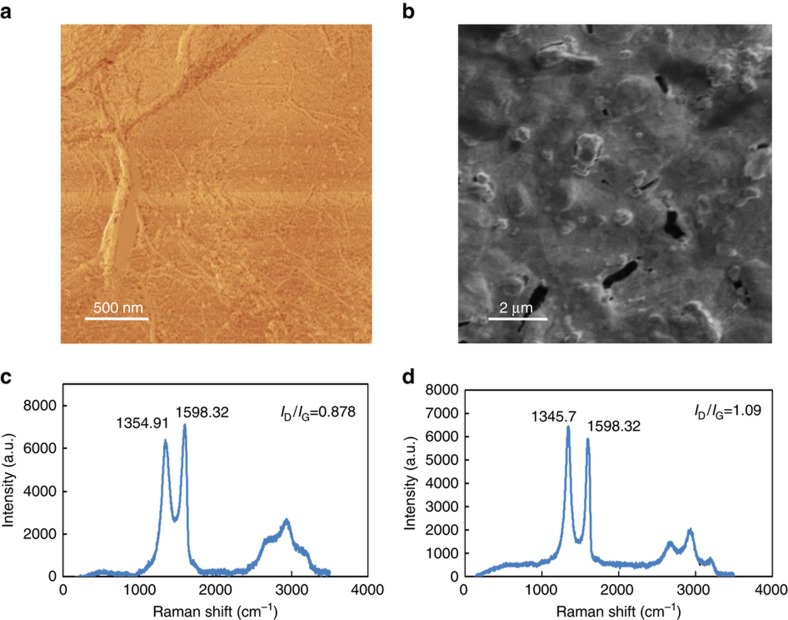
(**a**) Atomic force microscopy image of the GO layer on Si/SiO_2_. (**b**) SEM of the GO thin layer on a gold electrode surface. (**c**) Raman spectrum of GO. (**d**) Raman spectrum of rGO.

Figure 3a shows AFM images of GO deposited on a Si/SiO_2_ substrate. In most areas, there is a uniform flat GO layer with wrinkles and areas in which agglomeration has occurred. These can be produced during vaporization of the solvent, which can perhaps be avoided by using a lower concentration of GO solution or by drying the substrate in a vacuum. However, because our focus was on the electrochemical properties of GO and the sensitivity of the nitrite sensor (rather than the intrinsic electronic properties of GO), we intentionally did not remove these defects. In addition, the electrochemical edges of GO might be more sensitive than the flat regions. The effects of these defects on sensitivity should be systematically explored in future studies but were considered beyond the scope of this work. To ensure reproducible fabrication, the same concentrations and volumes and top glass slides of equal size were used. An advantage of using electrochemical graphene sensors is that we can obtain the active surface area from the slope of a plot of the current versus the square root of the scan rate and, instead of absolute current, the data can be calibrated and reported as current density. In this way, we can neglect possible differences between the coverages of graphene on the electrode surface. In addition, this film-coated glass slide method yields more uniform coatings than the usual drop cast method and avoids agglomeration, which can cause large differences in active surface area between electrodes.

Figure 3b shows a representative SEM image obtained from a larger area of the GO layer that was directly deposited on a gold electrode. This image shows that we were able to fully cover the surface uniformly, even on a gold working electrode with a surface roughness of several microns. The image also indicates that the number of stacked GO layers was minimized. The modified drop-cast method presented here is thus more suitable for obtaining larger areas of GO without agglomeration than regular drop casting.

The efficiency of the electrochemical reduction of GO was also investigated using Raman spectroscopy. Figure 3c shows the Raman spectrum of the GO substrate before reduction, and Figure 3d shows the Raman spectrum of the same substrate after 30 cycles of electrochemical reduction. The data shown represent the average of three measurements that were recorded at different areas/spots on each sample.

The main features observed in Raman spectra of carbon-based materials are the G and D peaks. These peaks arise from vibrations of sp^2^ carbons and appear at ~1580 and 1350 cm^−1^, respectively. The overtone of the D peak appearing at ~2700 cm^−1^ is termed the 2D peak. Unlike mechanically exfoliated graphene, GO is more disordered; therefore, the 2D band is usually of low intensity. Thus, GO and rGO can be distinguished based on the G and D peaks and the magnitude of the ratio of their intensities. In addition, the G peak of GO and rGO is shifted to higher frequencies (1598 cm^−1^) with respect to graphene and graphite due to the presence of defects. During the thermal reduction of GO, *I*_D_/*I*_G_ remains constant, although an increase in the *I*_D_/*I*_G_ ratio of rGO after electrochemical reduction has been reported in the literature^55^. In this study, this ratio was significantly increased compared to that for GO (from 0.87 to 1.1). This shows a restoration of sp^2^ carbons and a decrease in the average size of the sp^2^ domains after the electrochemical reduction of GO. An increase in the magnitude of the 2D peak also suggests enhanced graphitization^56^.

### Electrochemical detection of nitrite

**Figure 4:**
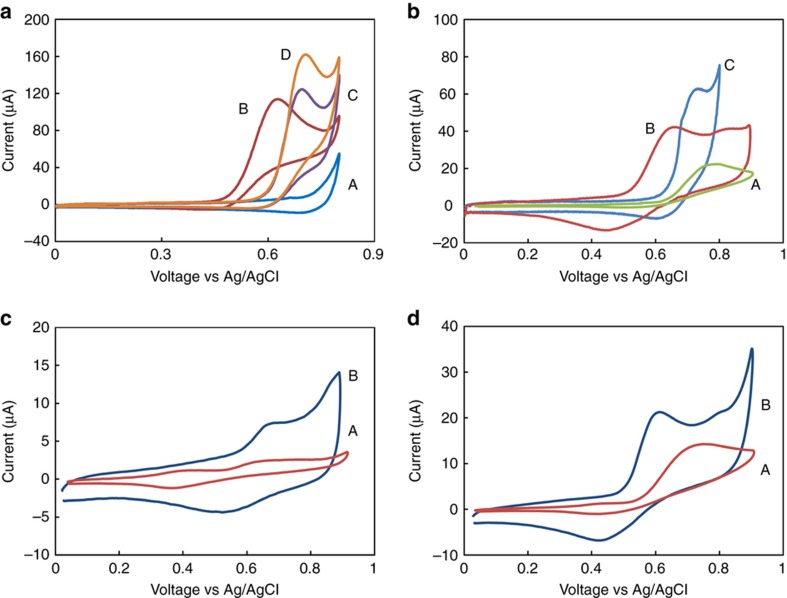
Current versus voltage curves obtained using (**a**) cyclic voltammetry and the rGO electrode. (A) PBS after washing three times; (B) 5 mM nitrite in acetate buffer, pH 6, (C) 0.1 M KCl, and (D) PBS buffer, pH 7.4. (**b**) Cyclic voltammetry measurements of 1 mM nitrite and (A) the gold electrode of SPE and rGO in (B) acetate buffer, pH 6, and (C) PBS buffer, pH 7.4, respectively. The voltage range is 0–0.9 V, and the scan rate is 50 mV s^−1^. (**c**–**d**) Cyclic voltammetry of (A) GO, (B) rGO in the presence of 100 μM and 1 mM nitrite. The scan rate is 25 mV s^−1^.

After fully reducing the GO electrode, we characterized the electrochemical performance of our platform for nitrite detection. Because both electrolyte identity and pH affect the sensitivity and detection limit of the sensor, we investigated the electrochemical response of the rGO sensor using cyclic voltammetry in various electrolytes. Figure 4a shows the oxidation peak of 5 mM nitrite in phosphate-buffered saline (PBS, pH 7.4), 0.1 M KCl, and acetate buffer (pH 6) as measured at a scan rate of 50 mV s^−1^. Anodic peaks appeared at 0.69, 0.7, and 0.63 V for the PBS, KCl, and acetate buffers, respectively. Given that the goal of this work is to develop a portable sensing platform that can operate under ambient conditions (in which O_2_ may react with the analyte), we avoided purging O_2_ in our samples. This enabled us to assess how the sensor will perform on real biological samples under ambient conditions. As seen from the voltammetric measurements conducted in EBC samples, the voltage of the oxidation peaks was shifted to positive voltages; this is more a favorable regime to use due to the lower over-potentials. We therefore opted to use the acetate buffer (pH 6) as the electrolyte for the remainder of the experiments. Another important factor in our deciding to use acetate was the fact that EBC samples from patients with inflammatory disease are reportedly acidic^26^. Thus, pH 6 more closely approximates the actual pH of EBC samples that would be obtained from patients with chronic inflammatory disease.

**Figure 5:**
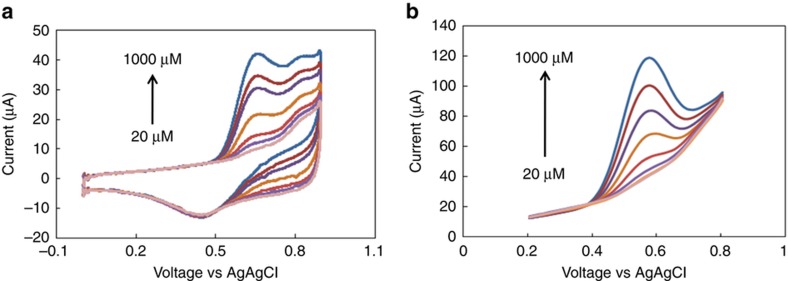
Current versus voltage curves obtained using (**a**) cyclic voltammetry of varying concentrations of nitrite from 20 to 1000 μM at pH 6 with a scan rate of 50 mV s^−1^. (**b**) Square wave voltammetry of varying concentrations of nitrite. Square wave voltammetry was also performed from 0 to 0.9 V with a step potential of 10 mV, an amplitude of 50 mV, and a frequency of 5 Hz.

The performance of the rGO-modified electrodes was compared to those of the SPE- and GO-deposited electrodes. Figure 4b shows a comparison between the anodic peaks in the presence of 1 mM nitrite for rGO electrodes at pH 6 and pH 7.4 and those for the SPE electrodes at pH 6 (50 mV s^−1^ scan rate). As clearly seen from the figure, rGO has a higher current and lower over-potential than the unmodified SPE electrode. Figures 4c and d also show the response of both the GO- and rGO-modified electrodes in the presence of 100–1000 μM nitrite, respectively (scan rate, 25 mV s^−1^). Figure 5 shows the result of cyclic voltammetry (Figure 5a) and square wave (Figure 5b) voltammetry analysis for nitrite concentrations from 20 to 1000 μM (scan rate, 50 mV s^−1^).

### Detection of nitrite in clinical EBC samples

After the performance of the fabricated sensor was confirmed in a standard electrolyte containing various concentrations of nitrite, we proceeded to test the graphene-based sensors in the complex biological matrix of EBC to study the effects of that biological matrix on the sensor. The results obtained provide insight into the response expected for clinical samples.

**Figure 6:**
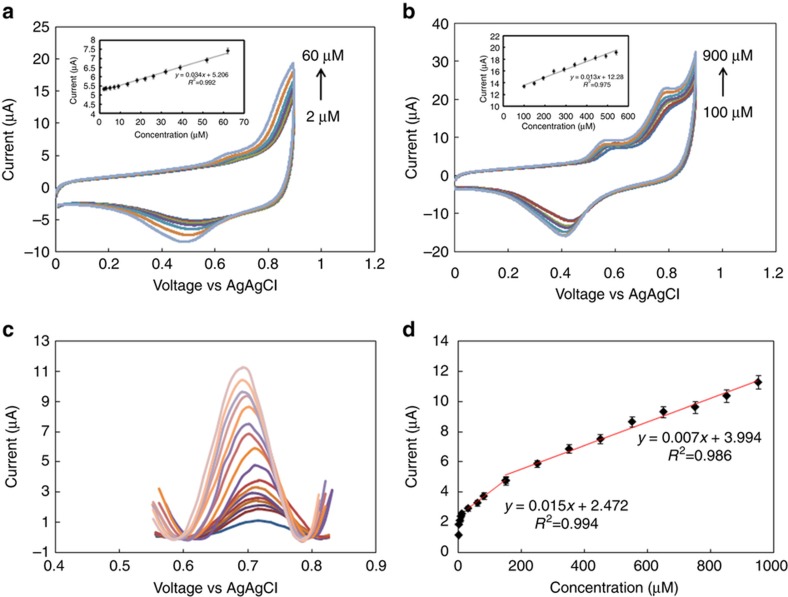
(**a**, **b**) Cyclic voltammogram of rGO electrodes at different nitrite concentrations ranging from 2 to 60 μM and 100 to 900 μM, which were spiked into the EBC samples (scan rate of 25 mV s^−1^). (**c**) Square wave voltammogram of spiked (concentration range from 0 to 1000 μM) EBC samples. The pulse amplitude is 50 mV. (**d**) Calibration curve showing the respective slopes.

Nitrite levels in EBC have been reported in the μM range^7,53^. We used both cyclic voltammetry (CV) and square wave voltammetry (SWV) to measure the redox current resulting from spiking buffer solutions with various concentrations of nitrite into the EBC sample. Figures 6a and b show the voltammetric response of the sensor to solutions containing 2–1000 μM nitrite at a scan rate of 25 mV s^−1^. The magnitude of the redox current generated for 1 mM nitrite was similar between the EBC and buffer solution matrices (Figure 6d). However, as previously mentioned, the potential in the EBC was shifted to a higher over-potential of 0.79 V. This can occur because the presence of proteins in EBC samples can slow electron transfer. The insets shown in Figures 6a and b are calibration curves based on CV measurements. The current response is linear in the concentration range of interest. We also studied the analytical performance of the nitrite sensor by taking SWV measurements conducted in the range of 0–0.9 V. Figure 6c displays the square wave voltammograms of nitrite in the range from 2 to 1000 μM. The redox current peak is found at ~0.7 V. Figure 6d shows the calibration curve obtained using SWV.

To consistently compare the performance of different sensors and account for device-to-device variations, we report sensitivity based on current density, which requires a knowledge of the active surface area of the electrodes. We used the Randles–Sevcik equation to calculate current density. We performed cyclic voltammetry on 5 mM K_3_Fe(CN)_6_ and plotted the peak current versus *ν*^1/2^. Based on the Randles–Sevcik equation, the relationship is linear, and thus the slope of the curve can be used to determine the active surface area. From the slope, we estimated the active surface area of the electrode to be ~0.07 cm^2^. This allows the sensitivity to be determined based on the surface area in the linear dynamic range. For EBC samples, the sensitivity is 0.21 μA μM^−1^ cm^−2^ in the range from 20 to 100 μM and the sensitivity is 0.1 μA μM^−1^ cm^−2^ in the range from 100 to 1000 μM. We determined the detection limit to be 830 nM based on three standard deviations. The detection sensitivity of nitrite in the presence of EBC is comparable with values reported in the literature for nitrite content in various buffers^45–47^.

**Figure 7:**
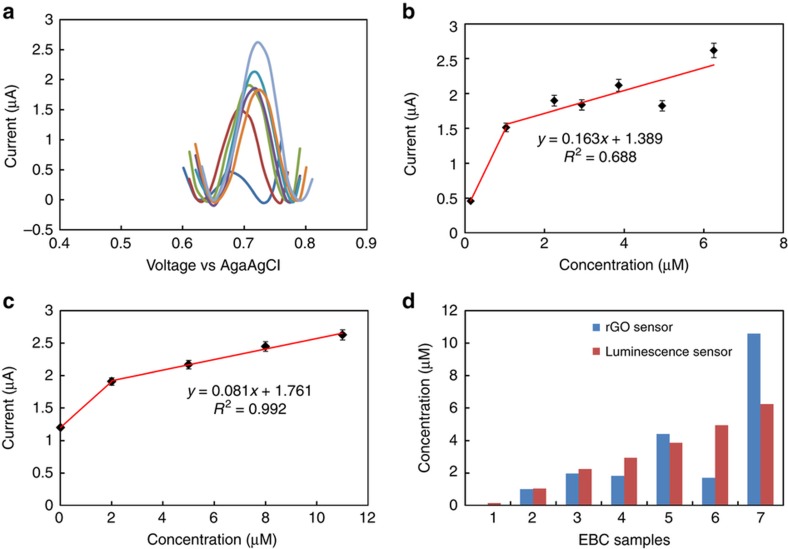
(**a**) Square wave voltammogram obtained for seven EBC samples. (**b**) The calibration curve was obtained based on results obtained using spiked samples, (**c**) a calibration curve based on chemiluminescence data, (**d**) a comparison between predicted concentration and chemiluminescence data.

After validating the functionality of the rGO nitrite sensor with spiked EBC samples, we proceeded to test the accuracy of the devices using a set of seven previously characterized clinical EBC samples. We performed SWV for each of the seven characterized samples (Figure 7a). The slight differences in oxidation potential between samples might be due to differences in the complex EBC matrix between individuals. The nitrite concentration in each sample was calculated from the measured oxidation currents based on the calibration data obtained using the spiked standard nitrite solutions in EBC (Figure 7b). We benchmarked the accuracy of our measurements by comparing the readings from our graphene-based sensor with measurements obtained using an ozone-based chemiluminescence technique^57^. Figures 7c and d show the results of this comparison. The range of measured nitrite concentrations based on the chemiluminescence experiments is 0.14–6.5 μM. For at least five of the seven samples, the relationship is linear, and strong agreement is found between the results obtained using the rGO sensor and the chemiluminescence measurements.

Regarding the two outlying data points (samples 6 and 7), more experimentation is necessary to understand the possible reasons behind their deviation. One possibility relates to the fact that the EBC samples were collected, frozen, and characterized by chemiluminescence several years prior to the electrochemical characterization experiments that were performed in this current study. This opens up the possibility that the nitrite content might have degraded over time in the frozen EBC samples (due to the possible conversion of nitrite to nitrate), thus highlighting the need for methods that can be used to measure samples at point-of-use immediately upon their collection from patients. In this experiment, acetate buffer (pH 6) was added to the EBC samples in a 1:1 ratio, and we assumed that the pH and conductivity of the samples were consistent between samples; however, it is possible that this assumption was not completely valid and that pH and conductivity might have varied between samples. Because the EBC sample volumes were small, we could not use a standard-sized pH meter electrode to measure pH; thus, we were unable to independently validate the consistency of pH and conductivity among the samples. To correct this problem, the integration of a microfabricated pH sensor and conductivity sensor on the same chip might provide insights that would allow more precise comparisons between samples.

## Conclusion

In this study, we utilized the outstanding properties of graphene-based electrodes to fabricate and characterize an enzyme-free sensor that proved capable of detecting nitrite samples in clinical samples of exhaled breath condensate. We also formed a micro-electrochemical cell using PDMS, which allowed us to perform measurements using small sample volumes of the target material (EBC). We systematically optimized various electrochemical parameters in buffer solution, thus allowing us to accurately measure nitrite in human EBC samples. The sensitivity of the nanofabricated sensor was 0.21 μA μM^−1^ cm^−2^ in the range from 20 to 100 μM and 0.1 μA μM^−1^ cm^−2^ in the range from 100 to 1000 μM with a detection limit of 830 nM. Most importantly, we were able to validate the performance of our sensors on clinical EBC samples that had been previously characterized using chemiluminescence. We demonstrated that the sensor exhibited high levels of precision in quantifying nitrite in the clinically relevant μM range. Future studies can be dedicated to fabricating sensors with improved detection limits using techniques, such as the use of nanoparticle-RGO composites and enzymatic modification of the electrodes, as well as improving the sensitivity of the electronic readout instrumentation.
